# Impact of Breast Cancer and Germline BRCA Pathogenic Variants on Fertility Preservation in Young Women

**DOI:** 10.3390/life13040930

**Published:** 2023-04-01

**Authors:** Elze Prokurotaite, Margherita Condorelli, Julie Dechene, Jason Bouziotis, Matteo Lambertini, Isabelle Demeestere

**Affiliations:** 1 Fertility Clinic, Department of Obstetrics and Gynecology, H.U.B—Hôpital Erasme, Université Libre de Bruxelles, 1070 Brussels, Belgium; 2 Research Laboratory on Human Reproduction, Université Libre de Bruxelles, 1070 Brussels, Belgium; 3 Department of Biomedical Research, H.U.B—Hôpital Erasme, Université Libre de Bruxelles, 1070 Brussels, Belgium; 4 Department of Internal Medicine and Medical Specialties (DiMI), School of Medicine, University of Genova, 16132 Genova, Italy; 5 Department of Medical Oncology, U.O. Clinica di Oncologia Medica, IRCCS Ospedale Policlinico San Martino, 16132 Genova, Italy

**Keywords:** fertility preservation, oocyte cryopreservation, ovarian reserve, BRCA mutation, germline BRCA pathogenic variants, breast cancer, preimplantation genetic diagnosis, pregnancy and follow-up

## Abstract

Background: Several studies have suggested that breast cancer (BC) and germline BRCA pathogenic variants (gBRCA PVs) could have a deleterious impact on ovarian reserve. Nevertheless, data are limited and mixed. Our objective was to evaluate the performance of fertility preservation (FP) in terms of the number of collected mature oocytes after ovarian stimulation (OS) in young women carrying a gBRCA PV, associated or not with BC. Methods: We conducted a retrospective monocentric study at HUB-Hôpital Erasme in Brussels. All women aged between 18 and 41 years diagnosed with invasive non-metastatic BC and/or gBRCA PV carriers who underwent OS for FP or preimplantation genetic testing for monogenic disorder (PGT-M) between November 2012 and October 2021 were included. Three groups were compared: BC patients without a gBRCA PV, BC patients with a gBRCA PV, and healthy gBRCA PV carriers. Ovarian reserve was evaluated based on the efficacy of OS and AMH levels. Results: A total of 85 patients underwent 100 cycles. The mean age (32.2 ± 3.9 years; *p* = 0.61) and median AMH level (1.9 [0.2–13] μg/L; *p* = 0.22) were similar between groups. Correlations between the number of mature oocytes and AMH level (*p* < 0.001) and between AMH and age (*p* < 0.001) were observed. No differences in the number of retrieved mature oocytes were observed between groups (*p* = 0.41), or for other OS parameters. Conclusion: Neither BC nor a gBRCA PV significantly affects ovarian reserve and FP efficacy in terms of the number of mature oocytes retrieved.

## 1. Introduction

Breast cancer (BC) is the most commonly diagnosed malignancy in premenopausal women [1], and the prevalence of BC is increasing in this population [2]. Approximately 10% to 15% of BC cases are inherited, and 60% to 80% of these carry a deleterious germline BRCA pathogenic variant (gBRCA PV) [3,4]. Using multigene panel analyses, other mutations conferring an increased risk of BC have been identified in less commonly mutated genes (TP53, PTEN, CDH1, MSH1, MLH1, MSH6, PMS2, PALB2, STK11, CHEK2, ATM, and BRIP1) [3]. 

Many young BC patients are candidates for (neo)adjuvant chemotherapy [5]. Given the gonadotoxicity of (neo)adjuvant chemotherapy regimens, fertility preservation (FP) is recommended in young patients diagnosed with BC [1,6,7]. Moreover, there is a global trend toward delaying childbearing, meaning that many patients may not have fulfilled their family planning goals at the time of diagnosis [8].

Different FP options are available, such as ovarian stimulation (OS) for oocyte/embryo cryopreservation and ovarian tissue cryopreservation. The administration of gonadotropin-releasing hormone (GnRH) agonists during chemotherapy can be an additional strategy to prevent premature ovarian insufficiency (POI) and increase the chances of pregnancy [9]. Oocyte/embryo cryopreservation is currently considered to be the standard of care for FP in BC patients when a delay of at least 12 days is acceptable before oncological treatment in order to perform OS and oocyte collection [2,10]. Healthcare providers should be prepared to discuss FP options and/or to refer all potential patients to specialized reproductive specialists as early as possible [2]. OS cycles can delay the onset of BC treatment [11], but it has been shown that this delay has no consequence in terms of oncological outcomes [12,13]. Ovarian tissue cryopreservation and/or in vitro maturation can be offered as alternatives when OS is not feasible due to a lack of time or to personal reasons. The use of GnRH agonists in association with (neo)adjuvant chemotherapy to preserve ovarian function should be discussed as a complementary FP option combined with a gamete cryopreservation procedure or in patients who are not interested in gamete cryopreservation but might benefit from a reduced risk of POI and increased chance for pregnancy [1]. 

Although oocyte or embryo cryopreservation is offered as the first option, there are still conflicting data regarding the expected response to OS for FP in BC patients. Cancer can be associated with increased catabolism and malnutrition, which could affect the hypothalamic–pituitary axis, and the occurrence of psychological stress could lead to an increase in prolactin and endogen opioid release, thus affecting the ovarian reserve [14]. The potential impact of cancer on gametes is well described in male patients, who often face a decreased quality and quantity of sperm prior to treatment. However, the impact of a cancer diagnosis on gonadal function in females is less clear [15].

OS protocols in BC patients usually include letrozole in order to decrease the high estrogen serum concentrations induced by gonadotrophins. Estrogen may cause the initiation, promotion, and progression of carcinogenesis in the breast by the metabolism of estrogen to genotoxic, mutagenic metabolites and the stimulation of tissue growth [16]. Letrozole is a highly selective aromatase inhibitor and is widely used in BC treatment [12,17]. Recent studies and meta-analyses did not report a difference in mature oocyte retrieval or in BC relapse rates with letrozole-associated OS compared to classic OS protocols [18,19,20,21]. 

BRCA1 and BRCA2 are tumor suppressor genes involved in the repair of double-strand DNA breaks [22]. They are principally involved in the DNA homologous repair pathway, where one strand of the sister chromatid serves as a template to recover the damaged sequence on the other strand [23]. This type of DNA repair can occur only in the G2 and S phases of the cell cycle. Briefly, the MRN complex recognizes the double-strand break and activates ATM by phosphorylation, which then activates a series of downstream effectors, including BRCA1. BRCA1 binds to PALB2 on the damaged DNA site and recruits BRCA2. BRCA2 binds RAD51, blocking its early DNA polymerization activity and allowing it to invade the template strand. As the repair process unwinds, the cell can progress in the cycle via p53 control [23].

Deleterious mutations in one of the BRCA genes may result in the accumulation of DNA damage. The most recognized intermediate step for cancerogenic transformation is the loss of heterozygosity of the BRCA genes, which mainly involves breast tissue and the ovaries [24]. The specific site predisposition seems to be linked to increased estradiol and progesterone levels in gBRCA PV carriers [25]. High progesterone serum levels increase the intake of RANKL in the breast tissue and decrease the circulating levels of osteoprotegerin (RANKL antagonist). The increased intake of RANKL contributes to mammary tumorigenesis, and low levels of osteoprotegerin are associated with increased proliferation of the breast tissue [25].

In gBRCA PV carriers, BC often occurs during the reproductive years [2]. BRCA1-mutated patients have a lifetime risk of developing BC of 65% and an ovarian cancer risk of 39%, while the risk of BC is 45% and the risk of ovarian cancer is 17% in BRCA2-mutated patients [26]. BRCA genes are essential to oocyte survival for preventing potential genetic stress; thus, any deleterious mutation may lead to accelerated loss of ovarian reserve [2]. gBRCA PV carriers require appropriate counseling and specific expertise in terms of FP [27]. Thus, FP can be considered in young women carrying a gBRCA PV even before the occurrence of BC if completion of childbearing before the recommended age for risk-reducing bilateral salpingo-oophorectomy is not feasible [28]. FP also offers the possibility to avoid transmission of the mutation after in vitro fertilization (IVF) using preimplantation genetic testing for monogenic disorder (PGT-M) to avoid the transmission of a pathogenic mutation to the offspring [29]. PGT-M is an in vitro method of embryo testing introduced in 1995 as an alternative to prenatal diagnosis potentially associated with the difficult decision of termination of pregnancy in case of an affected child. However, its usage in gBRCA PV carriers is a topic of debate due to the late onset, incomplete penetrance, and availability of preventive and therapeutic options. Its application is now recommended by guidelines and is actively applied in some countries such as the United Kingdom, Israel, The Netherlands, and Belgium [27,30].

A few studies have shown a significant association between gBRCA PV and decreased ovarian reserve in young BC patients [31]. In 2020, Porcu et al. reported that BRCA1-mutated patients had a diminished ovarian reserve (lower anti-Müllerian hormone (AMH) at baseline, higher dose of consumed gonadotropins, and lower number of cryopreserved oocytes) [32]. This reduced ovarian reserve puts them at a higher risk of developing POI after chemotherapy and reduces their chances of conception following treatment using their cryopreserved gametes [32,33,34]. In addition, Gunnala et al. in 2019 and Shapira et al. in 2015 did not detect any differences in AMH levels or OS outcomes in gBRCA PV carriers with or without BC [4,35]. Thus, considering the controversial evidence in the field, it is important to increase our knowledge on this topic, in order to better counsel patients on FP following gBRCA PV diagnosis and/or BC diagnosis. Several studies have used AMH values as a primary objective in order to assess ovarian reserve [32,36,37,38]. 

The primary objective of this study was to evaluate the performance of FP in terms of the number of collected mature oocytes from young gBRCA PV carriers, associated or not with BC, and compare it to that of mature oocyte retrieval from BC patients who do not carry a gBRCA PV. 

## 2. Materials and Methods

### 2.1. Study Design and Ethical Committee Approval

This was a monocentric retrospective study conducted at HUB-Hôpital Erasme in Brussels, Belgium. HUB-Hôpital Erasme is an FP reference center for around 15 oncological centers in Brussels, Wallonia, and Luxembourg. Data were retrieved from electronic medical files and registered in REDCap software (REDCap 10.0.20.). The general practitioners, gynecologists, and oncologists of the included patients were contacted in order to complete any missing information. The study was approved by the HUB-Hôpital Erasme ethics committee (protocol number: CE P2021/720).

All women aged between 18 and 41 years diagnosed with BC and/or as gBRCA PV carriers who underwent OS for FP or PGT-M between 29 November 2012 and 31 October 2021 were included in the study. The included patients were women newly diagnosed with invasive non-metastatic BC who underwent OS for FP or healthy women harboring a gBRCA PV who underwent OS for FP or PGT-M. All included patients had available gBRCA PV testing results and baseline AMH levels. 

Patients with a prior history of cancer and a prior history of gonadotoxic treatments, with metastatic (stage IV) BC at the time of diagnosis, with previous diagnosis of POI, or with prior infertility were excluded. Cycles cancelled for any reason (e.g., non-compliance with the OS protocol) were also excluded from analysis. We included only patients who did not express their refusal to participate in clinical studies.

### 2.2. Objectives

The primary objective was to evaluate the performance of FP in terms of the number of collected mature oocytes in young women diagnosed with a gBRCA PV, associated or not with BC. This cohort of patients with a gBRCA PV was compared to BC patients who did not carry a gBRCA PV. The secondary objectives were to compare ovarian reserve based on AMH levels, ovarian function based on hormone levels, oocyte maturation and fertilization rates, and oncological outcomes during follow-up.

### 2.3. Statistical Analysis

The mean and standard deviation were used to describe normally distributed data. The median and range were used to describe asymmetrical distributions. The comparison of means between groups was performed via ANOVA (if 3 independent groups were compared) or Student’s *t*-test (if 2 independent groups). The comparison of asymmetrical distributions was performed via the Kruskal–Wallis test or the Mann–Whitney–Wilcoxon test. Counts and percentages are presented for categorical data. Frequencies were compared between groups using Fisher’s exact test or Pearson’s Chi-squared, depending on the expected frequencies.

To compare data for OS and embryo transfer between groups, we used mixed-effects models with a random intercept at the patient level in order to take into account the correlation between observations of the same patient. We used mixed-effects multinomial regression if the outcome had more than 2 categories, mixed-effects logistic regression if the outcome was dichotomous, mixed-effects linear regression if the outcome was continuous, or mixed-effects Poisson or negative-binomial (if there was overdispersion) regression if the outcome was a count. 

The *p*-values of post hoc pairwise comparison tests after significant results were adjusted via the Bonferroni method.

The statistical significance was set at a *p* value of 0.05. The analyses were performed using Stata/IC 15.1.

## 3. Results

### 3.1. Study Population

**Figure 1:**
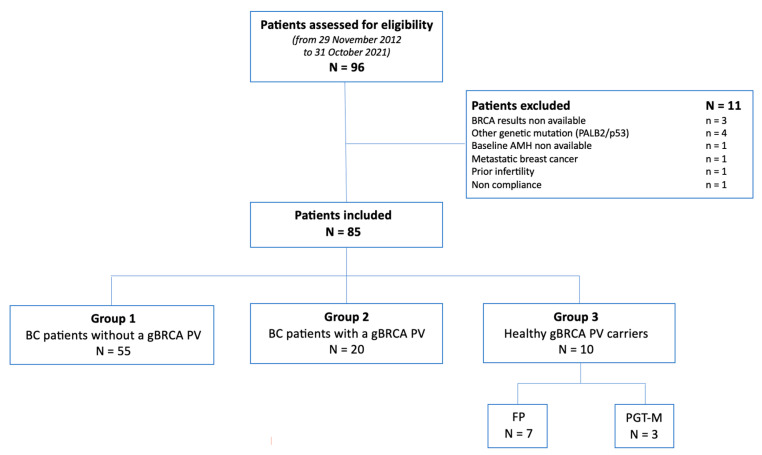
Flowchart for the study. Abbreviations: AMH: anti-Müllerian hormone; BC: breast cancer; gBRCA PV: germline BRCA pathogenic variant; FP: fertility preservation; PGT-M: preimplantation genetic testing for monogenic disorder.

We selected 96 patients and excluded 11 following the study eligibility criteria. A total of 85 patients were included in the 3 groups: (1) patients diagnosed with BC without a gBRCA PV, (2) patients diagnosed with BC with a gBRCA PV, and (3) healthy gBRCA PV carriers who underwent FP or PGT-M cycles (Figure 1).

Seventy-five patients (88.2%) were diagnosed with BC: fifty-five gBRCA PV-negative (64.7%) and twenty gBRCA PV-positive (23.5%). The other ten study participants (11.8%) were healthy carriers with a gBRCA PV: eight with an inherited BRCA1 mutation and two with a BRCA2 mutation. Seven and three of them underwent FP and IVF cycles with PGT-M, respectively (Table 1). 

The mean age of the cohort was 32.2 ± 3.9 years. No differences were observed in their reproductive history or hormonal contraception use (Table 1).

### 3.2. Breast Cancer

#### 3.2.1. Tumor Characteristics

There were no significant differences in the histology of cancer, tumor grade, tumor size, or nodal status between groups (Table 2). The majority of cases were diagnosed with a ductal carcinoma: 53 (96.4%) in Group 1 and 18 (90%) in Group 2. 

The majority of the cohort had high-grade tumors: 65.5% and 70% had grade III tumors in Group 1 and 2, respectively. Tumor size and nodal status were assessed using the international TNM classification. The T2 stage was predominant in both groups (49.1% in Group 1 and 80% in Group 2). The immunohistological tumor characteristics were statistically different between the groups (*p* = 0.01). The luminal B HER2-positive category was predominant in Group 1 (n = 18, 32.7%), and triple-negative tumors were predominant in Group 2 (n = 11, 55%) (Table 2). 

#### 3.2.2. Breast Cancer Treatment

BC patients with a gBRCA PV more often underwent mastectomy rather than conservative BC surgery as compared to the patients without a gBRCA PV (*p* = 0.002). Almost all of the patients received (neo)adjuvant chemotherapy (92.7% in Group 1 and 95% in Group 2). Anti-HER2 therapy was predominant in Group 1 (*p* = 0.02). As for other adjuvant treatments, there was no difference in radiotherapy or endocrine therapy between the groups (Table S1).

### 3.3. Ovarian Stimulation and Oocyte Retrieval

**Figure 2:**
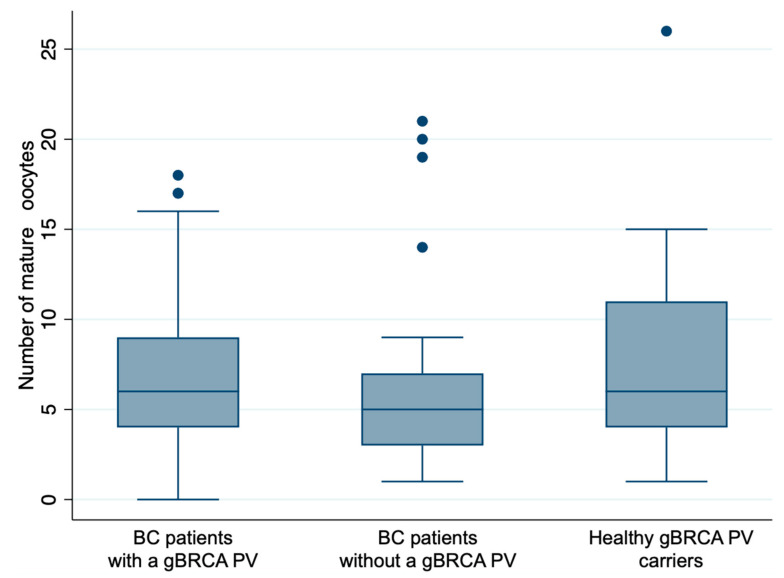
The distribution of the number of mature oocytes in the three study groups. No differences in mature oocyte retrieval were observed between the three groups (mixed-effects linear regression, *p* = 0.41). Abbreviations: BC: breast cancer; gBRCA PV: germline BRCA pathogenic variant.

A total of 85 patients underwent 100 cycles: 63 in Group 1, 22 in Group 2, and 15 in Group 3. There were three possible OS protocols according to the cycle phase at the time of starting OS by gonadotropins: standard (early follicular), random follicular, and random luteal, with no statistical differences between the groups. For BC patients, letrozole was systematically added to reduce estradiol (E2) levels during OS. There were no statistical differences between the groups in terms of the type of gonadotropin used, the total dose received, or the duration of OS. E2 and progesterone levels were similar at baseline. E2 levels were higher in healthy gBRCA PV carriers at triggering (adjusted *p* value < 0.001) compared to BC patients as they did not receive letrozole during OS. We observed no differences in the number of follicles punctured, the total number of oocytes, or mature oocytes collected between groups (Figure 2). The oocyte maturation rate was >80% in all groups. There were no differences in the number of cryopreserved oocytes or in the number of fertilized oocytes, with fertilization rates of over 70% in Groups 1 and 3 and 54.4% in Group 2 (Table 3).

### 3.4. Ovarian Reserve

The median baseline serum AMH was similar between groups (*p* = 0.22). The AMH value was inversely correlated with age (*p* < 0.001) (Figure S1). A direct correlation was observed between the number of mature oocytes and AMH (*p* < 0.001) (Figure S2). Additionally, an inverse correlation was observed between mature oocytes and age (*p* < 0.001) (Figure S3). 

### 3.5. Embryo Transfer and Pregnancy

In all, 15% of patients returned to the clinic for an embryo transfer: 18% in the first group and 10% in the second and third groups (Table 4). BC patients returned within 3 years, and healthy gBRCA PV carriers returned within 2 months. Some patients received an embryo transfer more than once; nevertheless, they were all single-embryo transfers at the time. We observed six live births in Group 1, one in Group 2, and one in Group 3 (Table 4).

### 3.6. Oncological Follow-Up

We report 98% follow-up in the first group and 90% in the gBRCA PV group (Table 5). In terms of the follow-up length, there was no difference between groups (*p* = 0.15). We observed five cases of relapse in Group 1 and two cases in Group 2, all loco-regional, and one death in Group 1 (Table 5).

## 4. Discussion

This monocentric hospital-based retrospective study over a period of 10 years aimed to evaluate the impact of BC and gBRCA PV on OS. More specifically, we compared BRCA BC patients with non-BRCA BC patients and healthy gBRCA PV carriers. This is an original approach compared to most of the available studies in the literature which used other cancer patients or elective freezing as controls. Indeed, this choice of control group in prior studies may have induced bias as it neglected gBRCA PV, other non-BC malignancies, population differences in age, previous infertility, and other potential confounding factors [4,32,37]. 

We aimed to investigate the impact on collected mature oocytes, as the main objective, and to evaluate the effectiveness of OS [39]. Although BC represents the main indication for FP, decreased ovarian reserve in women with gBRCA PV remains controversial (Table 6).

In case of BC patients or non-BC malignancies, we used AMH as an ovarian reserve marker. In healthy gBRCA PV carriers, OS for FP should be considered if completion of childbearing before the recommended age of risk-reducing bilateral salpingo-oophorectomy is not feasible for the potentially reduced ovarian reserve in these patients or for PGT-M before an embryo transfer [28]. All of these patients should be referred for counselling on FP and/or on PGT-M [27]. Previous studies mainly investigated ovarian reserve (AMH at baseline, dose of consumed gonadotropins, OS duration) and OS outcomes (total and mature oocyte retrieval, maturation and fertilization rates, number of cryopreserved oocytes and embryos). Fabiani et al. also studied oocyte quality, suggesting that BC might be associated with lower oocyte quality [40]. All studies compared different groups of patients: BC patients with versus without a gBRCA PV, BC patients versus patients with other malignancies, or healthy gBRCA PV carriers versus controls. Some of these studies concluded that BC patients with a gBRCA PV had a similar ovarian reserve and response to OS compared to gBRCA PV non-carriers or to controls [4,35,38]. However, others showed that patients with a gBRCA PV had a diminished ovarian reserve and/or lower numbers of mature oocytes [10,31], particularly for BRCA1 carriers [32,41] but also for BRCA2 carriers [37]. Two recent studies suggested that ovarian reserve was not diminished in BC patients with gBRCA PV as compared to BC patients without a gBRCA PV or to controls, but they also observed a lower response to OS in BC patients with gBRCA PV [42], as well as lower oocyte quality in BC patients [40]. Porcu et al. and Oktay et al. suggested that gBRCA PV carriers have a higher risk of POI due to decreased ovarian reserve at baseline [32,41].

As previously discussed, AMH reflects the ovarian reserve, decreases with age, and is also a useful marker of OS performance [43]. It has been hypothesized that gBRCA PVs may be responsible for lower AMH levels, suggesting a lower ovarian reserve due to defects in the repair of double-stranded DNA breaks [2]. However, we did not observe a difference in the median baseline serum AMH levels between groups (*p* = 0.22). As shown in Table 6, there are some discrepancies in the literature regarding the impact of gBRCA PVs on the ovarian reserve, but only a few studies have analyzed the difference between healthy gBRCA PV carriers and gBRCA PV patients with BC. Some authors included as a control group infertile women or non-BC malignancies [4,32,35,37,40]. This selection of controls could be a source of bias, as those patients were not screened for a gBRCA PV. In our study, all patients had a known gBRCA PV status. 

In accordance with our results on AMH levels, we did not observe any differences in the number of follicles punctured, the numbers of total and mature oocytes collected, or cryopreserved oocytes. These findings suggest that BC and gBRCA PV status do not affect the performance of FP. Importantly, we did not observe a difference between gBRCA PV carriers with or without BC, showing that the cancer did not increase the potential negative impact of the genetic pathogenic variants. Lambertini et al. observed a slightly lower number of oocytes in BC patients with a gBRCA PV as compared to BC patients without a gBRCA PV [10]. Porcu et al. and Oktay et al. both concluded that BC patients with BRCA1 mutation were at higher risk of POI, confirmed by a diminished ovarian reserve and a lower number of mature oocytes [32,41]. On the other hand, Gunnala et al. suggested that healthy gBRCA PV carriers had a comparable ovarian reserve and responses to OS to patients who had undergone elective egg freezing [4]. Shapira et al. also concluded that there was a normal ovarian response in IVF cycles in gBRCA PV patients, independent of BC status [35].

Importantly, maturation and fertilization rates appear to be similar in our cohort, suggesting no defects in oocyte quality, although our cohort is small and no data on embryo development were collected. Thus, these results should be confirmed in a large cohort, including pregnancy outcomes. 

In all, 10–20% of patients returned to the clinic for an embryo transfer: 18% in Group 1, 10% in Group 2, and 10% in Group 3. Ter Welle-Butalid et al. reported that 23% of BC patients returned for an embryo transfer after FP [44]. 

Nevertheless, the cohort remains too small to draw strong conclusions. Lambertini et al. observed a pregnancy rate at 10 years of 19% in BC survivors harboring a gBRCA PV, and they concluded that pregnancy after BC in gBRCA PV carriers is associated with favorable fetal outcomes and is safe from the oncological perspective [45].

Our study was conducted in a reference center where the treatment strategies are well known by all physicians, following recent recommendations. We chose the number of mature oocytes obtained during OS as a main indicator of fertility and oocyte competency. We collected other parameters that could influence fertility, such as age and AMH levels, to evaluate confounders. We evaluated the performance of FP in BC patients with or without a gBRCA PV and in healthy gBRCA PV carriers. Our study covers 10 years of observations, with an oncological follow-up and a small percentage of loss to follow-up. 

There are some limitations to our study, such as the limited number of patients included and the monocentric retrospective study design.

## 5. Conclusions

This study showed that BC patients without a gBRCA PV, BC patients with a gBRCA PV, and healthy gBRCA PV carriers had similar results in terms of mature oocyte retrieval following OS. The number of oocytes collected, which is a surrogate of ovarian reserve, was influenced only by AMH levels and patient age, not by the presence of gBRCA PVs. 

Larger multicenter studies are needed to confirm these preliminary data and address concerns regarding ovarian reserve, fertility, and pregnancy outcomes in gBRCA PV carriers.

## Figures and Tables

**Table 1:** Population characteristics (n = 85).

Population Characteristics	Group 1 BC Patients without a gBRCA PV n = 55 (64.7%)	Group 2 BC Patients with a gBRCA PV n = 20 (23.5%)	Group 3 Healthy gBRCA PV Carriers n = 10 (11.8%)	*p* Value	Statistical Test
Age at cryopreservation—mean ± SD	31.9 ± 4	32.8 ± 4	32.8 ± 3.6	0.61	ANOVA
BMI—mean ± SD	23.6 ± 4.1	24.5 ± 4.4	20.7 ± 3.4	0.054	ANOVA
Gestity—median [range]	0 [0–3]	0 [0–3]	0 [0–1]	0.58	Kruskal-Wallis
Parity—median [range]	0 [0–3]	0 [0–1]	0 [0–1]	1.00	Kruskal-Wallis
Hormonal contraception use—n (%)				0.16	Fisher’s exact
yes	23 (41.8)	8 (40)	7 (70)		
no	30 (54.6)	11 (55)	2 (20)		
unknown	2 (3.6)	1 (5)	1 (10)		

Abbreviations: BC: breast cancer; gBRCA PV: germline BRCA pathogenic variant; SD: standard deviation; BMI: body mass index.

**Table 2:** Breast cancer characteristics in the cohort of breast cancer patients (n = 75).

Breast Cancer Characteristics	Group 1 BC Patients without a gBRCA PV n = 55 (73.3%)	Group 2 BC Patients with a gBRCA PV n = 20 (26.7%)	*p* Value	Statistical Test
Age at BC diagnosis—mean ± SD	31.9 ± 4	32.8 ± 4	0.40	*T* test
Histology—n (%)			0.13	Fisher’s exact
Ductal carcinoma	53 (96.4)	18 (90)		
Lobular carcinoma	0	2 (10)		
Other	2 (3.6)	0		
Tumor grade—n (%)			0.61	Fisher’s exact
1	5 (9.1)	0		
2	13 (23.6)	6 (30)		
3	36 (65.5)	14 (70)		
Unknown	1 (1.8)	0		
Tumor size—n (%)			0.06	Pearson’s Chi-squared
T1	21 (38.2)	3 (15)		
T2	27 (49.1)	16 (80)		
T3–T4	7 (12.7)	1 (5)		
Nodal status—n (%)			0.85	Pearson’s Chi-squared
N0	37 (67.3)	13 (65)		
N1–N3	18 (32.7)	7 (35)		
Subtypes of BC—n (%)			0.01 *	Fisher’s exact
Luminal A	6 (10.9)	5 (25)		
Luminal B HER2 negative	11 (20)	3 (15)		
Luminal B HER2 positive	18 (32.7)	1 (5)		
HER2 positive	6 (10.9)	0		
Triple negative	14 (25.5)	11 (55)		

Abbreviations: BC: breast cancer; gBRCA PV: germline BRCA pathogenic variant; HER2: human epidermal growth factor receptor 2; ER: estrogen receptor; PR: progesterone receptor. * After correction of the *p* value for 10 post hoc pairwise comparisons, no difference remained significant.

**Table 3:** Ovarian stimulation and oocyte retrieval (n = 85).

	Group 1 BC Patients without a gBRCA PV n = 55 (64.7%)	Group 2 BC Patients with a gBRCA PV n = 20 (23.5%)	Group 3 Healthy gBRCA PV Carriers n = 10 (11.8%)	*p* Value	Statistical Test
**Baseline hormone level**—median [range]					
AMH (μg/L)	2.3 [0.3–13]	1.7 [0.2–4.7]	1.8 [0.5–8.3]	0.22	Kruskal-Wallis
FSH (IU/L)	5 [1–21]	6.2 [3–15]	5.5 [1–9.7]	0.15	Kruskal-Wallis
E2 (ng/L)	42 [12–499]	25 [16.5–135]	43 [16–289]	0.09	Kruskal-Wallis
**Number of cycles**	63	22	15		
Type of ovarian stimulation cycle—n (%)				0.47	ME multinomial R
Standard	31 (49.2)	14 (63.6)	11 (73.4)		
Random follicular	10 (15.9)	4 (18.2)	2 (13.3)		
Random luteal	22 (34.9)	4 (18.2)	2 (13.3)		
Gonadotropins				0.99	ME logistic R
Recombinant FSH—n (%)	51 (81)	19 (86.4)	10 (66.7)		
hMG—n (%)	12 (19)	3 (13.6)	5 (33.3)		
Total FSH dose (IU)—mean ± SD	2609.5 ± 1081	2717.6 ± 803.5	2451.7 ± 881	0.46	ME linear R
Total FSH dose (IU)—median [range]	2475 [25–5100]	2700 [1750–4200]	2400 [1250–4200]		
Stimulation (days)—median [range]	11 [3–17]	10 [6–14]	10 [6–14]		
Stimulation (days)—mean ± SD	10.4 ± 2.8	10.3 ± 2.6	9.7 ± 1.9	0.57	ME linear R
Trigger—n (%)				1.00	ME logistic R
hCG	13 (20.6)	7 (31.8)	8 (53.3)		
GnRH agonist	50 (79.4)	15 (68.2)	7 (46.7)		
Data at triggering—median [range]					
E2 (ng/L)	353.3 [55–1063]	287 [47–1345]	1604 [646–3733]	<0.001 *	ME linear R
Progesterone (μg/L)	1 [0.3–5.7]	0.8 [0.3–2.4]	1 [0.3–2.6]	0.08	ME linear R
Number of follicles > 18 mm	2 [0–11]	2 [0–6]	2 [1–6]	0.28	ME linear R
Number of follicles 15–18 mm	4 [0–20]	3 [0–17]	4 [0–22]	0.25	ME linear R
Number of follicles < 15 mm	6 [0–24]	7 [1–10]	7 [1–19]	0.17	ME linear R
**OS outcomes**					
Number of oocytes collected—median [range]	8 [0–21]	6 [1–22]	8 [1–29]	0.36	ME linear R
Number of oocytes collected—mean ± SD	8.9 ± 5,2	7.7 ± 6.2	9.3 ± 6.9		
Number of mature oocytes collected—median [range]	6 [0–18]	5 [1–21]	6 [1–26]	0.41	ME linear R
Number of mature oocytes collected—mean ± SD	7.2 ± 4.5	6.9 ± 6.1	8 ± 6.2		
Maturation rate (%)—mean ± SD	81.0 ± 20.7	86.2 ± 19.9	89.4 ± 17.1	0.26	ME linear R
Total number of cryopreserved oocytes	325	88	80		
Number of cryopreserved oocytes—median [range]	5 [0–17]	3 [0–21]	1 [0–26]	0.98	ME negative binomial R
**Fertilization outcomes**					
Total number of oocytes fertilized	125	28	40		
Number of oocytes fertilized—median [range]	2 [1–12]	2 [1–12]	5 [3–11]	0.10	ME Poisson R
Fertilization rate (%)—mean ± SD	70.4 ± 37.4	54.4 ± 40.2	73.2 ± 22.2	0.45	ME linear R
Total number of cryopreserved embryos	85	13	9		
Number of cryopreserved embryos—median [range]	2 [0–9]	1.5 [1–3]	2 [0–3]	0.03 **	ME Poisson R

Abbreviations: BC: breast cancer; gBRCA PV: germline BRCA pathogenic variant; OS: ovarian stimulation; AMH: anti-Müllerian hormone; FSH: follicle-stimulating hormone; LH: luteinizing hormone; hMG: human menopausal gonadotropin; IU: international units; SD: standard deviation; hCG: human chorionic gonadotropin; GnRH: gonadotropin-releasing hormone; E2: estradiol; ME R: mixed-effects regression. * Group 1 versus Group 2: adjusted *p* value = 1.00; Group 1 versus Group 3: adjusted *p* value < 0.001; Group 2 versus Group 3: adjusted *p* value < 0.001.** Group 1 versus Group 2: adjusted *p* value = 0.165; Group 1 versus Group 3: adjusted *p* value = 0.135; Group 2 versus Group 3: adjusted *p* value = 1.00.

**Table 4:** Descriptive data on embryo transfers and pregnancy outcomes (n = 85).

	Group 1 BC Patients without a gBRCA PV n = 55 (64.7%)	Group 2 BC Patients with a gBRCA PV n = 20 (23.5%)	Group 3 Healthy gBRCA PV Carriers n = 10 (11.8%)	*p* Value	Statistical Test
Patients returned to clinic for embryo transfer—n (%)	10 (18.2)	2 (10)	1 (10)	0.16	Fisher
Time elapsed from cryopreservation to return to clinic (years)—median [range]	2.2 [0.2–6.4]	2.9 [1.5–6.4]	0.2	0.19	Kruskal-Wallis
Embryo transfers—n	15	1	7		NA *
Pregnancy rate—n (%)	10 (66.7)	1	2 (28.6)	0.11	ME logistic R
Pregnancy outcome—n					
Livebirth	6	1	1		NA *
Ongoing pregnancy	1	0	0		NA *
Spontaneous abortion	3	0	1		NA *
Patients with a livebirth—n	6	1	1		NA *

Abbreviations: BC: breast cancer; gBRCA PV: germline BRCA pathogenic variant; SD: standard deviation; ME R: mixed-effects regression; NA: not applicable. * Numbers were too low to reach statistical significance.

**Table 5:** Oncological follow-up of breast cancer patients (n = 75).

Oncological Follow-Up	Group 1 BC Patients without a gBRCA PV n = 55 (73.3%)	Group 2 BC Patients with a gBRCA PV n = 20 (26.7%)	*p* Value	Statistical Test
Patients with cancer follow-up—n (%)	54 (98.2)	18 (90)	0.17	Fisher’s exact
Cancer follow-up (months)—median [range]	42 [3–98]	45 [13–95]	0.15	Mann-Whitney-Wilcoxon
Relapse—n (%)	5 (9.3)	2 (11.1)	1.00	Fisher’s exact
Type of relapse			1.00	Fisher’s exact
Loco-regional	5	2		
Metastatic	0	0		
Death—n	1	0	1.00	Fisher’s exact

Abbreviations: BC: breast cancer; gBRCA PV: germline BRCA pathogenic variant.

**Table 6:** Studies investigating ovarian reserve in women with gBRCA PV with or without breast cancer.

References	Number of Patients	Type of Study	Groups	Objective	Main Findings
Fabiani et al. 2022 [40]	294	monocentric retrospective case-control	BC patients (n = 105) Controls (n = 189)	Impact of BC on the ovarian response and the oocyte quality	BC does not seem to be associated with a lower ovarian reserve but is linked with worsening oocyte quality
Sung Woo et al. 2022 [42]	117	multicenter retrospective	BC patients with a gBRCA PV (n = 39) BC patients without a gBRCA PV (n = 20) BC patients with unknown gBRCA PV status (n = 58)	Impact of the gBRCA PV and hormone receptor status on ovarian reserve and OS outcomes in BC patients	BC patients with aBRCA PV have comparable ovarian reserve but a lower response to OS (*p* = 0.002)
Porcu et al. 2020 [32]	227	monocentric prospective	BC patients with BRCA1 (n = 11) BC patients with BRCA2 (n = 11) BC patients without a gBRCA PV (n = 24) Controls (n = 181)	Impact of BRCA1 and BRCA2 on ovarian reserve and FP outcomes	BRCA1 is associated with a higher risk of POI confirmed by a diminished ovarian reserve and a lower number of mature oocytes (*p* < 0.05)
Son et al. 2019 [31]	316	monocentric retrospective	BC patients with a gBRCA PV (n = 52) BC patients without a gBRCA PV (n = 264)	Association between gBRCA PV and AMH	BC patients with a gBRCA PV have significantly lower serum AMH level (*p* = 0.004)
Gunnala et al. 2019 [4]	795	retrospective	BC patients with a gBRCA PV (n = 38) BC patients without a gBRCA PV (n = 53) Non-BC malignancies (n = 85) Healthy gBRCA PV carriers (n = 19) Controls (n = 600)	Impact of gBRCA PV and malignancy (BC and non-BC) on ovarian reserve	gBRCA PV carriers with and without malignancy have comparable ovarian reserve and responses to OS
Lambertini et al. 2018 [10]	156	retrospective	BC patients with a gBRCA PV (n = 29) BC patients without a gBRCA PV (n = 72) BC patients with unknown gBRCA PV status (n = 55)	Impact of gBRCA PV on AMH and performance of FP	BC patients with a gBRCA PV have a consistent trend for reduced reproductive potential and performance of FP (*p* > 0.05)
Johnson et al. 2017 [37]	195	prospective	BRCA1 carriers (n = 55) BRCA2 carriers (n = 50) gBRCA PV non carriers (n = 26) Controls (n = 64)	Association between a gBRCA PV and AMH	BRCA2 carriers have a significantly lower AMH levels incompared to low-risk controls (*p* = 0.021)
van Tilborg et al. 2016 [38]	255	multicenter prospective	Healthy gBRCA PV carriers (n = 124) Healthy gBRCA PV non carriers (n = 131)	Association between a gBRCA PV and AMH	gBRCA PV carriers do not show a lower serum AMH level in comparison to proven non-carriers (*p* = 0.34)
Shapira et al. 2015 [35]	124	multicenter retrospective	BC patients with a gBRCA PV (n = 21) BC patients without a gBRCA PV (n = 21) gBRCA PV carriers (n = 41) gBRCA PV non carriers (n = 41)	Impact of gBRCA PV on performance of FP	Both BC patients with a gBRCA PV and healthy gBRCA PV carriers have a normal ovarian response in IVF cycles (OY *p* = 0.49, PRR *p* = 1)
Oktay et al. 2010 [41]	82	prospective	BC patients with a gBRCA PV (n = 14) BC patients without a gBRCA PV (n = 33) BC patients with unknown gBRCA PV status (n = 35)	Association between gBRCA PV and performance of FP	BRCA1 is associated with an occult POI (OY *p* = 0.025, PRR *p* = 0.014)

Abbreviations: BC: breast cancer; gBRCA PV: germline BRCA pathogenic variant; DFS: disease-free survival; FP: fertility preservation; POI: premature ovarian insufficiency; AMH: anti-Müllerian hormone; OS: ovarian stimulation; IVF: in vitro fertilization; OY: oocyte yield; PRR: poor response rate.

## Data Availability

Data are available upon reasonable request.

## Supplementary Materials

The following supporting information can be downloaded at: https://www.mdpi.com/article/10.3390/life13040930/s1, Table S1. Breast cancer treatment. Figure S1. The relationship between AMH and age. Figure S2. The relationship between number of mature oocytes and AMH. Figure S3. The relationship between number of mature oocytes and age.
