# Supplementary material for: Impact of Breast Cancer and Germline BRCA Pathogenic Variants on Fertility Preservation in Young Women
Source: Life (Basel). 2023 Apr 1;13(4):930. doi: 10.3390/life13040930 (PMC10146760; doi:10.3390/life13040930)
Supplement: Supplementary file 1 [file life-13-00930-s001.zip › life-2257375-supplementary.pdf]

Supplementary Data

Table S1. Breast cancer treatment (n = 75).

Breast Cancer Treatment	Group 1 BC Patients without a gBRCA PV n = 55 (73.3%)	Group 2 BC Patients with a gBRCA PV n = 20 (26.7%)	*p* Value	Statistical Test
Breast surgery			0.002*	Pearson's Chi-squared
Tumorectomy—n (%)	36 (65.5)	5 (25)		
Mastectomy—n (%)	19 (34.5)	15 (75)		
Axillary surgery			0.49	Fisher's exact
None—n (%)	1 (1.8)	0		
SLNB—n (%)	27 (49.1)	7 (35)		
ALND—n (%)	27 (49.1)	13 (65)		
Chemotherapy—n (%)	51 (92.7)	19 (95)	1.00	Fisher
Anti-HER2 therapy—n (%)	24 (43.6)	1 (5)	0.002	Pearson's Chi-squared
Radiotherapy—n (%)	49 (89.1)	14 (70)	0.11	Fisher's exact
Endocrine therapy—n (%)	34 (61.8)	9 (45)	0.19	Pearson's Chi-squared

Abbreviations: BC: breast cancer; gBRCA PV: germline BRCA pathogenic variant; SLNB: sentinel lymph node biopsy; ALND: axillary lymph node dissection; HER2: human epidermal growth factor receptor 2. * Significantly more mastectomies than tumorectomies in Group 2.

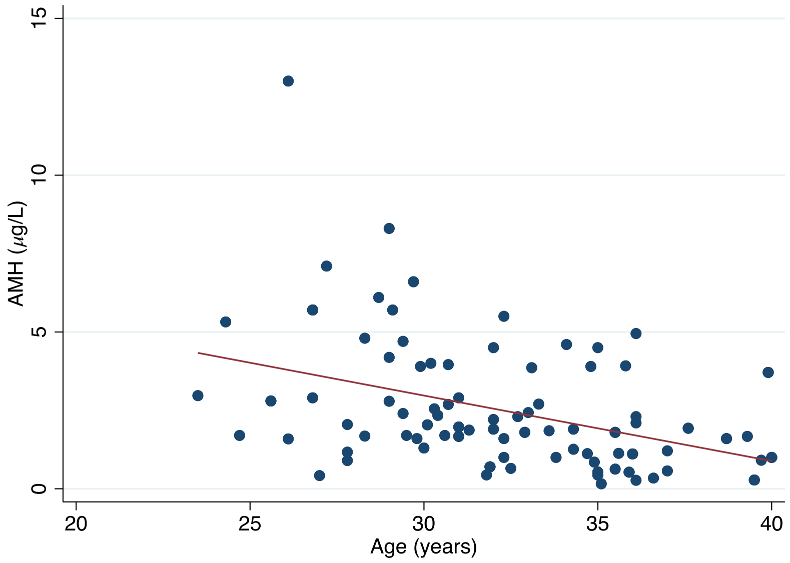

Figure S1. The relationship between AMH and age.
P < 0.001 by linear regression.

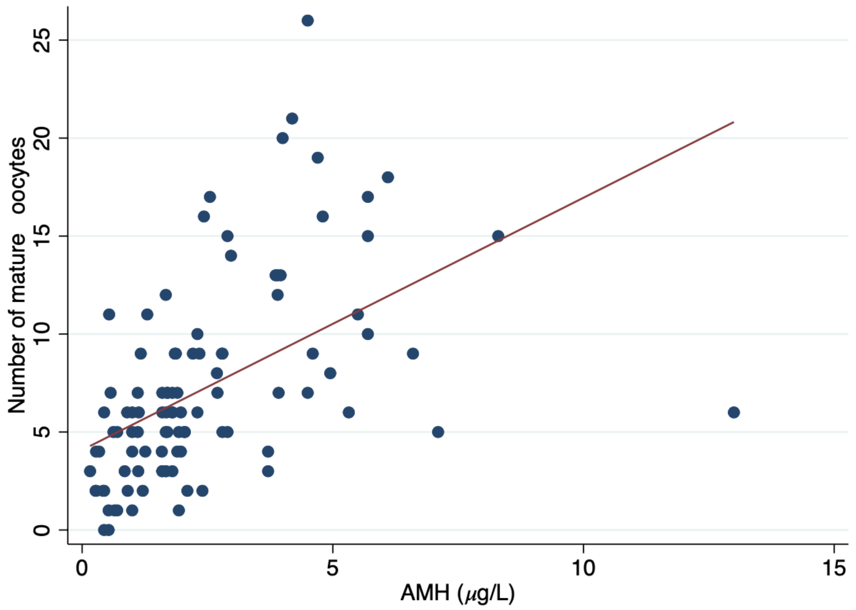

Figure S2. The relationship between number of mature oocytes and AMH.
P < 0.001 by mixed-effects linear regression.

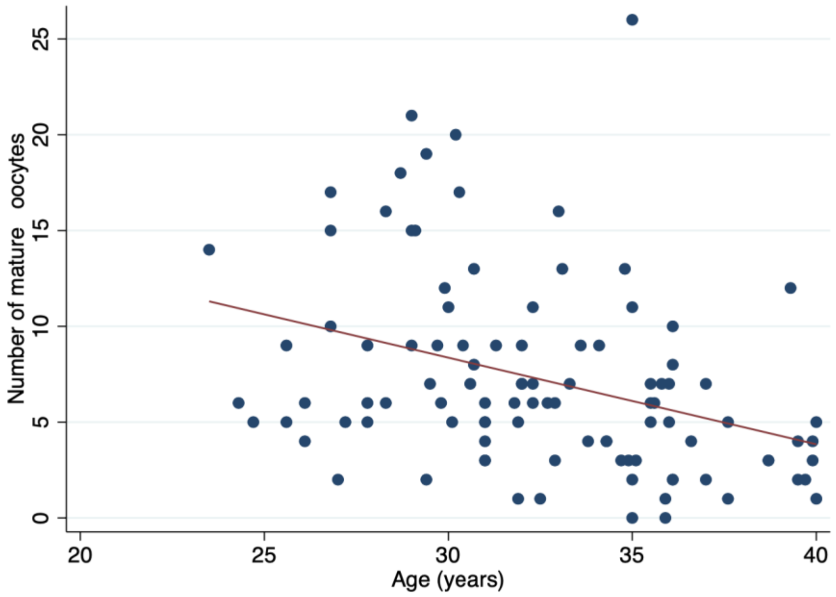

Figure S3. The relationship between number of mature oocytes and age.
P = 0.001 by mixed-effects linear regression.
